# Dynamic mechanism of eliminating COVID-19 vaccine hesitancy through web search

**DOI:** 10.3389/fpubh.2023.1018378

**Published:** 2023-01-30

**Authors:** Yixue Xia, Qifeng Li, Wei Jiao, Yuexin Lan

**Affiliations:** Research Center of Network Public Opinion Governance, China People's Police University, Langfang, China

**Keywords:** vaccine hesitancy, COVID-19 vaccination, web search, eliminating mechanism, Logistic model

## Abstract

This research focuses on the research problem of eliminating COVID-19 vaccine hesitancy through web search. A dynamic model of eliminating COVID-19 vaccine hesitancy through web search is constructed based on the Logistic model, the elimination degree is quantified, the elimination function is defined to analyze the dynamic elimination effect, and the model parameter estimation method is proposed. The numerical solution, process parameters, initial value parameters and stationary point parameters of the model are simulated, respectively, and the mechanism of elimination is deeply analyzed to determine the key time period. Based on the real data of web search and COVID-19 vaccination, data modeling is carried out from two aspects: full sample and segmented sample, and the rationality of the model is verified. On this basis, the model is used to carry out dynamic prediction and verified to have certain medium-term prediction ability. Through this research, the methods of eliminating vaccine hesitancy are enriched, and a new practical idea is provided for eliminating vaccine hesitancy. It also provides a method to predict the quantity of COVID-19 vaccination, provides theoretical guidance for dynamically adjusting the public health policy of the COVID-19, and can provide reference for the vaccination of other vaccines.

## 1. Introduction

Novel coronavirus was officially named 2019 coronavirus disease (COVID-19) by the World Health Organization in 2020. In the field of infectious disease prevention and control, vaccination is an effective measure to prevent and control the epidemic, and is one of the most cost-effective public health measures (1–5). In the prevention and control of COVID-19 epidemic, vaccination is also one of the priority measures taken by public health departments. However, in the process of COVID-19 vaccination, the phenomenon of “vaccine hesitancy” is widespread all over the world, which affects the public's acceptance of vaccines. Vaccine hesitancy refers to delay in acceptance or refusal of vaccination despite the availability of vaccination services (6), which has become one of the biggest obstacles to build the immune barrier of the population. How to eliminate COVID-19 vaccine hesitancy and increase the quantity of vaccination has become an urgent problem to be solved in building a group immune barrier. The important causes of vaccine hesitancy are summarized by WHO as three factors: confidence, complacency and convenience, in which the confidence refers to the confidence of vaccine safety, effectiveness and vaccination service; Complacency refers to the lack of awareness of the harm of diseases and doubts about the necessity of vaccination; Convenience refers to vaccine supply, acceptance of vaccine price, and accessibility of vaccination services (6).

**Figure 1:**
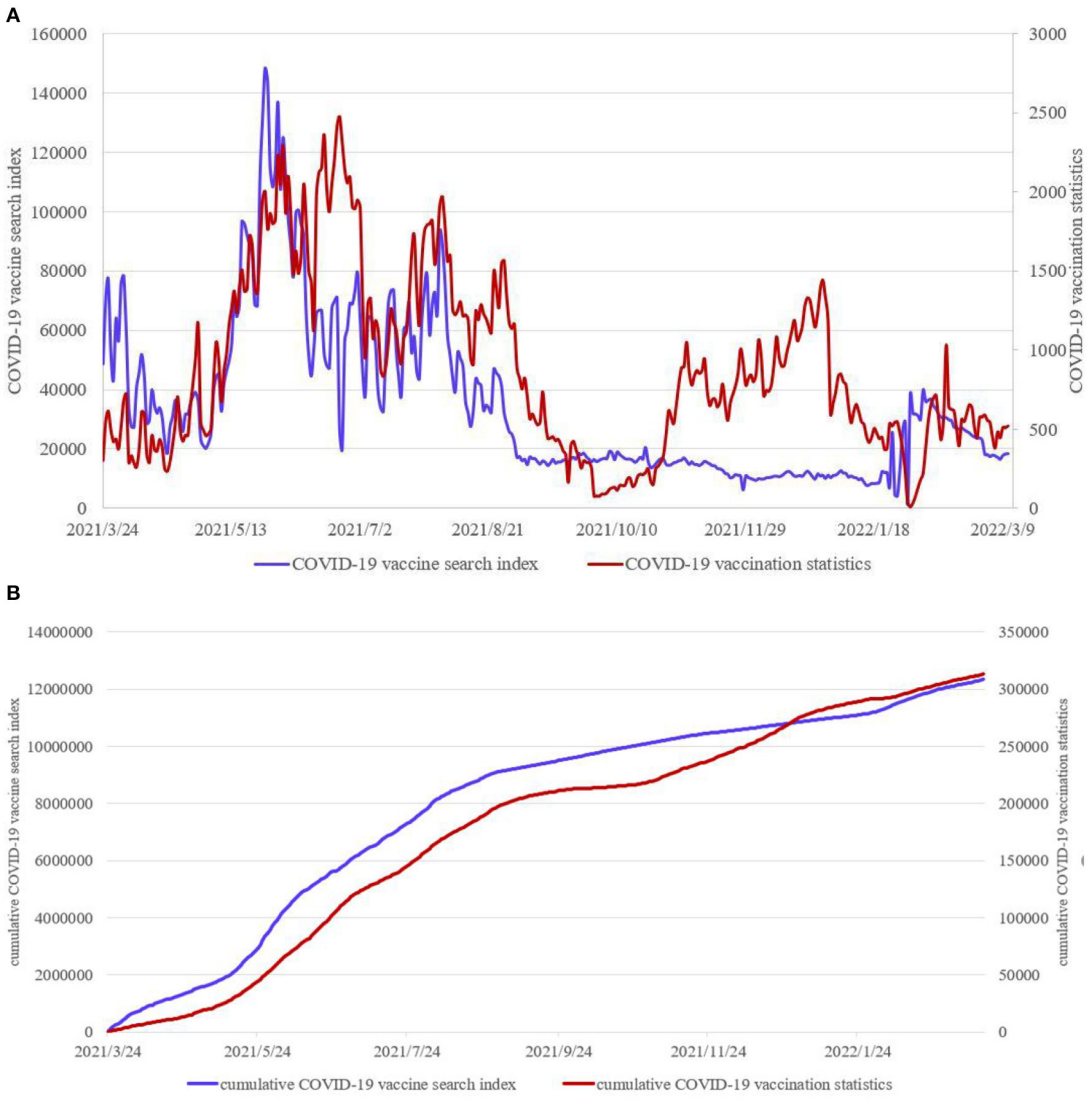
**(A)** COVID-19 vaccination data and Baidu Search Index data. **(B)** Corresponding cumulative data.

For the causes of “vaccine hesitancy”, governments and various media often make scientific and rational voices overcome other voices through science popularization and mobilization, so as to dispel the concerns and hesitations of some people and make “vaccination should be done and vaccination should be done as much as possible” become a reality. However, compared with this method of passively accepting propaganda to eliminate vaccine hesitancy, people in the Internet Age tend to prefer active web search to eliminate vaccine hesitancy and take the initiative to get vaccinated. So what is the relationship between web search data and vaccination data? On March 23, 2021, China's National Health Commission (http://www.nhc.gov.cn) began to announce the vaccination situation of COVID-19 in China, and updated the cumulative reported vaccination data of COVID-19 all over the country every day (http://www.nhc.gov.cn/xcs/yqjzqk/list_gzbd.shtml). In this research, we select COVID-19 vaccine vaccination data (unit: 10,000 doses) and Baidu Search Index data with COVID-19 vaccine as the key word from March 24, 2021 to March 13, 2022 to draw a comparison chart (https://index.baidu.com/v2/index.html#/) (Figure 1). Based on the search volume of netizens in Baidu and taking keywords as the statistical object, the Search Index is obtained by scientifically analyzing and calculating the weighted search frequency of each keyword in Baidu web search.

**Figure 2:**
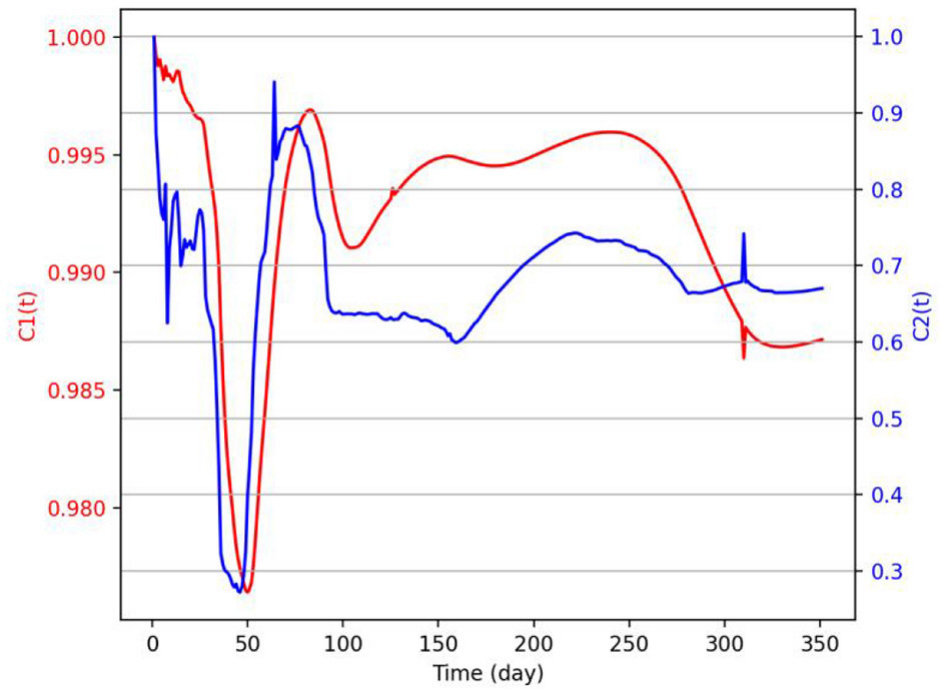
Dynamic correlation coefficient *c*1(*t*) and *c*2(*t*).

In order to explore the relationship between the two groups of data, this research has conducted a dynamic correlation analysis on COVID-19 vaccination data and web search data of COVID-19 vaccine. Calculate the dynamic correlation coefficient *c*1(*t*) between the cumulative data of the Baidu Search Index and the cumulative data of COVID-19 vaccination, and the dynamic correlation coefficient *c*2(*t*) between the Baidu Search Index data per day and the data of COVID-19 vaccination per day. The mean value of *c*1(*t*) is 0.99, and the mean value of *c*2(*t*) is 0.68, indicating that there is a strong correlation between the web search data and the COVID-19 vaccination data, especially the dynamic correlation between the cumulative data (Figure 2). Therefore, it is necessary to explore the correlation between the two variables by taking the cumulative quantity of COVID-19 vaccination data and the cumulative quantity of web search data as variables.

On this basis, this research assumes that web search will play a positive role in eliminating COVID-19 vaccine hesitancy and promoting COVID-19 vaccination. Then, a model is constructed to quantify the influencing mechanism of web search data on COVID-19 vaccination data, infer the degree of eliminating vaccine hesitancy, predict the vaccination trend through web search data, and propose some suggestions to eliminate COVID-19 vaccine hesitancy by promoting web search.

This research focuses on the problem of eliminating COVID-19 vaccine hesitancy through web search. The significance of this research mainly includes two aspects. First of all, the COVID-19 epidemic is still developing globally, and the uncertainty caused by the virus mutation makes the prevention and control of COVID-19 epidemic still worthy of attention. A new “multinational Delphi consensus to end the COVID-19 public health threat” pointed out that the government, public welfare organizations, enterprises and vaccine manufacturers should increase investment to develop vaccines against multiple SARS CoV-2 virus variants to provide lasting protection. At the same time, vaccine hesitancy remains a major challenge. In order to reduce vaccine hesitancy and improve vaccination rate, experts suggested that the government should take appropriate measures according to the local culture and the rejected population to improve vaccination rate (7). Therefore, exploring the feasibility of promoting the elimination of vaccine hesitancy through web search is of positive significance for the prevention and control of COVID-19 epidemic both in China and around the world, as well as for the elimination of vaccine hesitancy for other infectious diseases.

Secondly, based on the correlation between the COVID-19 vaccination data and web search data, this research constructed a model to quantify this correlation and explain the influencing mechanism of web search data on COVID-19 vaccination data. The model essentially describes the influence of web search on offline activities, and can be used as a basic model to study the influence of web search on other offline activities. For example, study how to promote the vaccination of other diseases with similar process to COVID-19 vaccination. Further, on the basis of this model, add or delete variables and parameters, or update the meaning of variables and parameters, to study the influence of web search on some offline activities. Therefore, the significance of this research is also to provide a new way to study the relationship between web search and offline activities, which has certain universal applicability.

The structure of this paper is as follows. Section 2 is a review of related research. Section 3 is modeling. In this section, a new dynamic model of eliminating COVID-19 vaccine hesitancy through web search is constructed based on the Logistic model, the degree of eliminating COVID-19 vaccine hesitancy through web search is quantified, the elimination function is defined to analyze the dynamic elimination effect, and the model parameter estimation method is proposed. Section 4 is simulation. The numerical solution, process parameters, initial value parameters and stationary point parameters of the model are simulated respectively, and the mechanism of elimination is deeply analyzed to determine the key time period. It is concluded that the role of the growth rate of web search is significantly better than the initial state of web search, and it is necessary to improve the degree of converting the quantity of web search into the quantity of COVID-19 vaccination. Section 5 is empirical analysis. Based on the real data of web search and COVID-19 vaccination, data modeling is carried out from two aspects: full sample and segmented sample, and the rationality of the model is verified. On this basis, the model is used to dynamically predict the COVID-19 vaccination trend and verified to have certain medium-term prediction ability.

## 2. Related work

### 2.1. Vaccine hesitancy

The initial research on vaccine hesitancy mainly focused on introducing the phenomenon of vaccine hesitancy, defining vaccine hesitancy, and summarizing the causes and determinants of vaccine hesitancy. By analyzing the scope and determinants of vaccine hesitancy, it is complex and specific, varying with time, place and vaccines (6). Personal decisions about vaccination are complex, involving emotional, cultural, social, spiritual and political factors as well as cognitive factors (8). Besides, some studies recognize a continuum between the domains of vaccine acceptance and vaccine refusal and de-polarize previous characterization of individuals and groups as either anti-vaccine or pro-vaccine (9).

Since then, much of the research on vaccine hesitancy has focused on specific groups and different regions. Young people in Japan are more hesitant about vaccines than the elderly. The degree of vaccine hesitancy in young women is significantly higher than that in young men, and the difference between the age groups in young men is higher than that in young women (10). In China, non-medical personnel, adults who have been vaccinated against influenza and old people have a low degree of hesitation about vaccination (11). Compared with medical students, most non-medical students are hesitant to receive COVID-19 vaccine (12). Parental hesitation about HPV vaccination is common in the United States, which is closely related to both insufficient vaccination and vaccine refusal (13, 14). COVID-19 vaccine hesitancy is common among pregnant and postpartum individuals (15). However, among pregnant women in Malaysian cities, the prevalence of vaccine hesitancy is relatively low. Muslim mothers are unlikely to hesitate about the vaccine (16). The rate of vaccine hesitancy in high-income countries or regions ranges from 7 to 77.9%. Forty-six studies (47.4%) have a ratio of 30% or more (17). Vaccine hesitancy is particularly prevalent among the wealthier groups, but the relationship between socioeconomic status and vaccine hesitancy in Low- and Middle-Income Countries (LMICs) is less clear. In addition, there is no significant correlation between education level and vaccine hesitancy (18).

Among them, there are also some studies on the measurement method of vaccine hesitancy and the analysis of survey results. The SAGE (the Strategic Advisory Group of Experts) Vaccine Hesitancy Working Group has developed a matrix of the determinants of vaccine hesitancy informed by a systematic review of peer reviewed and gray literature, and by the expertise of the working group. According to the definition of vaccine hesitancy and the determinant matrix, a set of survey questions to investigate vaccine hesitancy is set up to guide the development of a tool to assess the nature and scale of vaccine hesitancy (19). The VHS (Vaccine Hesitancy Scale) is tested in rural and urban Guatemala. The results show that there are problems in the interpretation of the VHS, especially in the presence of vaccine shortages and using a Likert scale that does not resonate across diverse cultural settings (20). In addition, studies have shown that greater hesitancy, both in general and specific to the influenza vaccine, is associated with lower vaccine uptake (21). Compared with COVID-19 vaccine promotion posts accompanied by pro-vaccine comments, those accompanied by anti-vaccine comments provoke greater reactance in audiences through the mediating effects of bandwagon perception and the presumed influence of the posts on others, and the greater reactance, in turn, increase audiences' COVID-19 vaccine hesitancy (22).

There is a lot of research on how to eliminate vaccine hesitancy, based on the definition of vaccine hesitancy and the characteristics and determinants of vaccine hesitancy in different populations. Studies have shown that dialogue-based interventions are most effective in correcting misconceptions and publicizing the safety and importance of vaccines to eliminate vaccine hesitancy (23, 24). Meanwhile, some research focuses on negative emotions such as fear and anxiety to stimulate positive emotions in people with vaccine hesitancy (25). Health care providers and the medical community are encouraged to adopt a multi-level strategy and effectively “Tweet up” to counter the growing threat posed by vaccine misinformation and hesitancy (26). Suggestions for adjusting the organizational strategy of contradictory information to control its popularity from different dimensions, such as poster's influence, activity and identity, tweets' topic, sentiment, readability are proposed, to reduce vaccine hesitancy (11).

In the above studies on vaccine hesitancy, the internal differences of individuals or groups and the external environment are the influencing factors of vaccine hesitancy. In the studies on eliminating vaccine hesitancy, methods such as dialogue, stimulating positive emotions, and adjusting social media publicity strategies have been discussed. In the context of the interaction between the Internet and offline society and the increasing influence of the Internet on offline society, promoting dialogue, stimulating emotions and carrying out publicity through the Internet is a noteworthy direction in the research of eliminating vaccine hesitancy. In addition, people in the Internet Age tend to prefer active web search to eliminate vaccine hesitancy and take the initiative to get vaccinated. Therefore, this research proposes the problem of eliminating vaccine hesitancy and realizing active vaccination through web search, which enriches the research topic of eliminating vaccine hesitancy through the Internet, and also provides a new idea for this field.

### 2.2. Web search

Web search data has been widely used to predict user behavior or event development. The researchers believe that the search terms people type represent their immediate needs and reflect the users themselves (27–30). There are relatively many studies using web search data to predict the occurrence of influenza or other diseases in the future. A digital syndromic surveillance system is developed based on social media and search queries data, experiments show that search engine data/Twitter have a significant temporal relationship with influenza and MERS data (31). Other studies have shown that online search query data is related to the number of new cases of AIDS (32), syphilis (33), and COVID-19 (34, 35). Therefore, web search query data can be used to predict the number of new cases of AIDS, syphilis, COVID-19, etc. Studies have shown that Internet searches and social media data have high correlation with daily incidences, with the maximum *r* > 0.89 in all correlations. The lag correlations also show a maximum correlation at 8–12 days for laboratory-confirmed cases and 6–8 days for suspected cases (34). Using Baidu search index as the data source, it is found that daily growth of confirmed cases and Baidu index values for each COVID-19-related symptom present robust positive correlations during the outbreak (35). In addition to the prediction of future influenza or disease, web search data can also be used to predict social and economic activities or public events such as unemployment rate (36), stock market (37), commodity prices (38), traffic conditions (39), tourism trends (40), etc.

The correlation between web search and offline activities is confirmed through the relevant researches on predicting social economic activities or public activities through web search data. How web search affects offline activities, what is its dynamic mechanism, and how to enhance or reduce this influence are worthy of in-depth study. With the development of network society, the web search data can better reflect the views, attitudes and behaviors of users. It also needs to be explored in depth not only in prediction, but also in promoting the transformation of users' views, attitudes and practical behaviors through web search. Through the literature review, it is found that there are relevant studies on predicting vaccination uptake using web search queries (41), but few studies specifically discuss the role of web search in eliminating vaccine hesitancy. This research proposes a new perspective of eliminating vaccine hesitancy through web search, and constructs a new model to explain its dynamic process. It is a new progress in the research of eliminating vaccine hesitancy and the application of web search.

In general, this research has made new progress in the following aspects: (1) Based on the Logistic model, a new model is constructed to describe the dynamic mechanism of eliminating COVID-19 vaccine hesitancy through web search. (2) By quantifying the elimination degree and simulating different parameters, the methods of eliminating vaccine hesitancy are enriched, and a new practical idea is provided for eliminating vaccine hesitancy. (3) Through the prediction research, this research provides a method to predict the quantity of COVID-19 vaccination, provides theoretical guidance for dynamically adjusting the public health policy of the COVID-19, and can provide reference for the vaccination of other vaccines.

## 3. Modeling

### 3.1. Modeling variables

**Figure 3:**
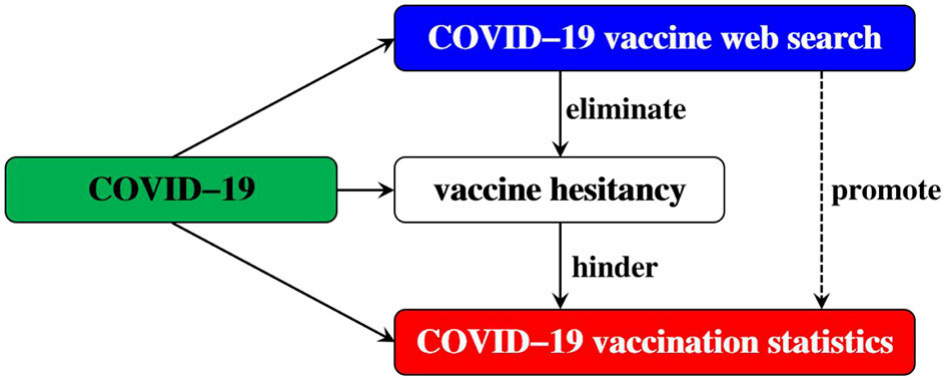
The process of eliminating COVID-19 vaccine hesitancy through web search.

After the outbreak of COVID-19, vaccination has become one of the most effective intervention measures, and it has been accompanied by COVID-19 vaccine hesitancy and web search with COVID-19 as the key word. Among them, vaccine hesitancy will hinder COVID-19 vaccination, while web search will eliminate vaccine hesitancy and promote COVID-19 vaccination (Figure 3). Based on this, this research chooses the quantity of COVID-19 vaccination as the core variable to study the dynamic mechanism of web search to eliminate COVID-19 vaccine hesitancy.

Considering that COVID-19 vaccination and its corresponding web search come into being with the COVID-19 epidemic, and experience the evolution process of occurrence, development and stability, this research uses the Logistic model to study the dynamic law of vaccination quantity and its corresponding web search quantity. As the main model in the field of quantitative sociodynamics, the Logistic model and innovation models based on Logistic model are widely used in population ecology, economics, information science and other fields. On the basis of mathematical analysis and calculation of Logistic model, scholars have extended it to multi-component systems, such as epidemic spread (42–46), information diffusion (47–51), etc., to study the evolution trend of data in multi-component systems, deal with the current situation reasonably, and make scientific predictions for the future.

Assuming that *x*(*t*) is the monotonically increasing quantity of COVID-19 vaccination and *s*(*t*) is the monotonically increasing quantity of COVID-19 vaccine web search. Then, according to the Logistic modeling idea, we get the following model


(1) $$\{\begin{array}{c} \frac{dx}{dt}=rx\left(1-\frac{x}{K}\right)\\ \frac{ds}{dt}=r_{1}s\left(1-\frac{s}{K_{1}}\right) \end{array}$$

The growth rate of COVID-19 vaccination quantity is *r, r*>0, the initial value is *x*(0), and the upper limit is *K*. The growth rate of COVID-19 vaccine web search quantity is *r*_1_, *r*_1_>0, the initial value is *s*(0), and the upper limit is *K*_1_. The Logistic model is used to describe the evolution process of things in a cycle, so in a cycle of co evolution of web search quantity and COVID-19 vaccination quantity, there is an evolution upper limit for both. It is easy to calculate and get the analytical solutions of the model as follows


(2) $$\{\begin{array}{c} x\left(t\right)=\frac{K}{1+(\frac{K}{x\left(0\right)}-1)\exp(-rt)}\\ s\left(t\right)=\frac{K_{1}}{1+(\frac{K_{1}}{s\left(0\right)}-1)\exp(-r_{1}t)} \end{array}\;$$

### 3.2. Influence of web search on COVID-19 vaccination quantity

After eliminating vaccine hesitancy through web search of COVID-19 vaccine, the quantity of COVID-19 vaccination will increase appropriately. Assuming that within Δ*t*, the quantity of web search per unit can increase the quantity of COVID-19 vaccination by $\alpha \frac{x}{K}$, then when the quantity of web search is $\frac{s\left(t\right)}{K_{1}}$, the remaining space for the increase of COVID-19 vaccination quantity changes from $\left(1-\frac{x}{K}\right)$ to $\left(1-\frac{x}{K}+\alpha \frac{s}{K_{1}}\frac{x}{K}\right)$, where α is the promoting coefficient. Therefore, the dynamic model of COVID-19 vaccination quantity change under the effect of web search is obtained.


(3) $$\{\begin{array}{c} \frac{dx}{dt}=rx\left(1-\frac{x}{K}+\alpha \frac{s}{K_{1}}\frac{x}{K}\right)\\ \frac{ds}{dt}=r_{1}s\left(1-\frac{s}{K_{1}}\right) \end{array}$$

Factor out the common factor get


(4) $$\{\begin{array}{c} \frac{dx}{dt}=rx\left(1-(1-\alpha \frac{s}{K_{1}})\frac{x}{K}\right)\\ \frac{ds}{dt}=r_{1}s\left(1-\frac{s}{K_{1}}\right) \end{array}$$

The specific meanings of variables and parameters in the model are shown in Table 1.

**Table 1:** Meaning of model variables and parameters.

**Variable or parameter**	**Meaning**	**Value range**
*x*(*t*)	Quantity of COVID-19 vaccination	0 ≤ *x* ≤ *K*
*x*(0)	Initial value of COVID-19 vaccination quantity	*x*(0)≥0
*s*(*t*)	Quantity of web search	0 ≤ *s* ≤ *K*_1_
*s*(0)	Initial value of web search quantity	*s*(0)≥0
*r*	Growth rate of COVID-19 vaccination quantity	*r*≥0
*K*	Upper limit of COVID-19 vaccination quantity	*K*>0
α	Promoting coefficient	α≥0
*r*_1_	Growth rate of web search quantity	*r*_1_≥0
*K*_1_	Upper limit of web search quantity	*K*_1_>0

Let


$$\{\begin{array}{c} \frac{dx}{dt}=rx\left(1-(1-\alpha \frac{s}{K_{1}})\frac{x}{K}\right)=0\\ \frac{ds}{dt}=r_{1}s\left(1-\frac{s}{K_{1}}\right)=0 \end{array}$$

The non-zero equilibrium point of the model is $\left(\frac{K}{1-\alpha },K_{1}\right)$, and the stability condition of the equilibrium point is 0 ≤ α < 1. The analytical solution of $\frac{ds}{dt}=r_{1}s\left(1-\frac{s}{K_{1}}\right)$ is $s\left(t\right)=\frac{K_{1}}{1+\left(\frac{K_{1}}{s\left(0\right)}-1\right)\exp\left(-r_{1}t\right)}$, so we can get that the analytical solution of the model (4) is


(5) $$x\left(t\right)=\frac{x(0)\exp(rt)}{1+x(0)\int_{0}^{t}\frac{rexp(rv)}{g(v)}dv}$$

Among them $g\left(v\right)=K/\left(1-\frac{\alpha }{1+\left(\frac{K_{1}}{s\left(0\right)}-1\right)\exp\left(-r_{1}v\right)}\right)$.

### 3.3. Quantify the degree of eliminating COVID-19 vaccine hesitancy through web search

According to the model (4), the equilibrium point changes from *K* to $\frac{K}{1-\alpha }$ after web search eliminates vaccine hesitancy, then *H*_∞_ is defined as the elimination rate of vaccine hesitancy through COVID-19 vaccine web search so as to analyze the future trend of eliminating COVID-19 vaccine hesitancy.


(6) $$\begin{array}{l} H_{\infty }=\frac{K-\frac{K}{1-\alpha }}{K}=\frac{\alpha }{1-\alpha } \end{array}$$

It is easy to see that the elimination rate *H*_∞_ is proportional to the promoting coefficient α. The larger the α, the greater the elimination rate, and the better the elimination effect. *x*(*t*) is changed from $\frac{K}{1+\left(\frac{K}{x\left(0\right)}-1\right)\exp\left(-rt\right)}$ to $\frac{x(0)\exp(rt)}{1+x(0)\int_{0}^{t}\frac{rexp(rv)}{g(v)}dv}$, so the elimination function of vaccine hesitancy through COVID-19 vaccine web search is defined as follows


(7) $$H\left(t\right)=(\frac{x(0)\exp(rt)}{1+x\left(0\right)\int_{0}^{t}\frac{rexp\left(rv\right)}{g\left(v\right)}dv}-\frac{K}{1+\left(\frac{K}{x\left(0\right)}-1\right)\exp\left(-rt\right)})/K$$

get


(8) $$H\left(t\right)=\frac{\exp(rt)}{\frac{K}{x(0)}+\int_{0}^{t}rf\left(v\right)\exp\left(rv\right)dv}-\frac{\exp(rt)}{\frac{K}{x\left(0\right)}+\exp(rt)-1}$$

where $f\left(v\right)=1-\frac{\alpha }{1+\left(\frac{K_{1}}{s\left(0\right)}-1\right)exp\left(-r_{1}v\right)}$ to analyze the dynamic elimination effect of vaccine hesitancy through COVID-19 vaccine web search. Because $\alpha \frac{s}{K_{1}}\frac{x}{K}\geq 0$, and *s*(*t*), *x*(*t*) are positive monotone increasing functions, we can get that $x\left(t\right)\geq \frac{K}{1+\left(\frac{K}{x\left(0\right)}-1\right)\exp\left(-rt\right)}$. *H*(*t*) is a non-negative monotone increasing function, so $0\leq H\left(t\right)<\frac{\alpha }{1-\alpha }$ considering $\underset{\tau \to +\infty }{\lim}H\left(t\right)=H_{\infty }=\frac{\alpha }{1-\alpha }$. Since the n-th derivative corresponding to *H*(*t*) is complex, it can be obtained by numerical calculation method according to specific parameters when calculating the n-th derivative.

In order to evaluate the dynamic elimination effect, and determine the promotion degree of eliminating vaccine hesitancy through web search and the degree of stimulating web search, it needs to analyze the calculation method of promoting coefficient α and growth rate of web search quantity *r*_1_ when the expected elimination effect needs to be achieved.

At time *t*_0_, in order to make the elimination function $H\left(t_{0}\right)\geq \bar{H}$ (expected value), we can get


(9) $$H\left(t_{0}\right)=\frac{\exp(rt_{0})}{\frac{K}{x(0)}+\int_{0}^{t_{0}}rf\left(v\right)\exp\left(rv\right)dv}-\frac{\exp\left(rt_{0}\right)}{\frac{K}{x\left(0\right)}+\exp\left(rt_{0}\right)-1}\geq \bar{H}$$

It can be obtained by sorting out the above formula


(10) $$\frac{\exp(rt_{0})}{\bar{H}+\frac{\exp\left(rt_{0}\right)}{\frac{K}{x\left(0\right)}+\exp\left(rt_{0}\right)-1}}-\frac{K}{x\left(0\right)}\geq \int_{0}^{t_{0}}rf\left(v\right)\exp\left(rv\right)dv$$

that is


(11) $$\int_{0}^{t_{0}}rf\left(v\right)\exp\left(rv\right)dv\leq \frac{\exp\left(rt_{0}\right)}{\bar{H}+\frac{\exp\left(rt_{0}\right)}{\frac{K}{x\left(0\right)}+\exp\left(rt_{0}\right)-1}}-\frac{K}{x\left(0\right)}$$

Where $\frac{\exp\left(rt_{0}\right)}{\bar{H}+\frac{\exp\left(rt_{0}\right)}{\frac{K}{x\left(0\right)}+\exp\left(rt_{0}\right)-1}}-\frac{K}{x\left(0\right)}$ is a constant. Therefore, at time *t*_0_, in order to make the elimination function $H\left(t_{0}\right)\geq \bar{H}$ (expected value), $\int_{0}^{t_{0}}rf(v)\exp(rv)dv\leq \frac{\exp(rt_{0})}{\bar{H}+\frac{\exp(rt_{0})}{\frac{K}{x(0)}+\exp(rt_{0})-1}}-\frac{K}{x(0)}$ is required. Because $\int_{0}^{t_{0}}rf(v)\exp(rv)dv$ is affected by α and *r*_1_, when α is fixed, the value range of *r*_1_ required to reach $\bar{H}$ can be calculated. When *r*_1_ is fixed, the value range of α required to reach $\bar{H}$ can be calculated.

### 3.4. Model parameter estimation

Change model (4) into its corresponding difference equation


(12) $$\{\begin{array}{l} \Delta x\left(k\right)=rx\left(1-\left(1-\alpha \frac{s}{K_{1}}\right)\frac{x}{K}\right)=rx-\frac{r}{K}x^{2}+\frac{r\alpha }{K_{1}K}sx^{2}\\ \Delta s\left(k\right)=r_{1}s\left(1-\frac{s}{K_{1}}\right)=r_{1}s-\frac{r_{1}}{K_{1}}s^{2} \end{array}$$

where Δ*x*(*k*) = *x*(*k*)−*x*(*k*−1), Δ*s*(*k*) = *s*(*k*)−*s*(*k*−1), *k* = 1, 2, 3…. It is observed that there is a ternary linear function relationship between Δ*x*(*k*) and *x, x*^2^, *sx*^2^, and a binary linear function relationship between Δ*s*(*k*) and *s, s*^2^. Therefore, model parameters *r, K*, α, *r*_1_, *K*_1_ can be obtained by multiple regression analysis on the premise that vaccination statistics and web search statistics are known.

## 4. Simulation

In the model (4), the parameters α and *K* are stationary point parameters, that is, affecting the equilibrium point of the model. When α and *K* are fixed, *r, r*_1_ *and K*_1_ are the process parameters, that is, affecting the changing process of model solution. *x*(0), *s*(0) are the initial value parameters. Therefore, we focus on the model solution, process parameter *r*_1_, initial value parameter *s*(0), and stationary point parameter α for numerical simulation, to study the dynamics of the change in the quantity of COVID-19 vaccination and the effect of web search on eliminating vaccine hesitancy. For the model (4), numerical simulation is carried out by taking any value within the range of parameter variation. Set the model parameters for *r* = 0.12, *K* = 1000, *x*(0) = 0.01*K*, *r*_1_ = 0.05, *K*_1_ = 500, *s*(0) = 0.01*K*_1_, α = 0.6.

### 4.1. Simulation of numerical solution

**Figure 4:**
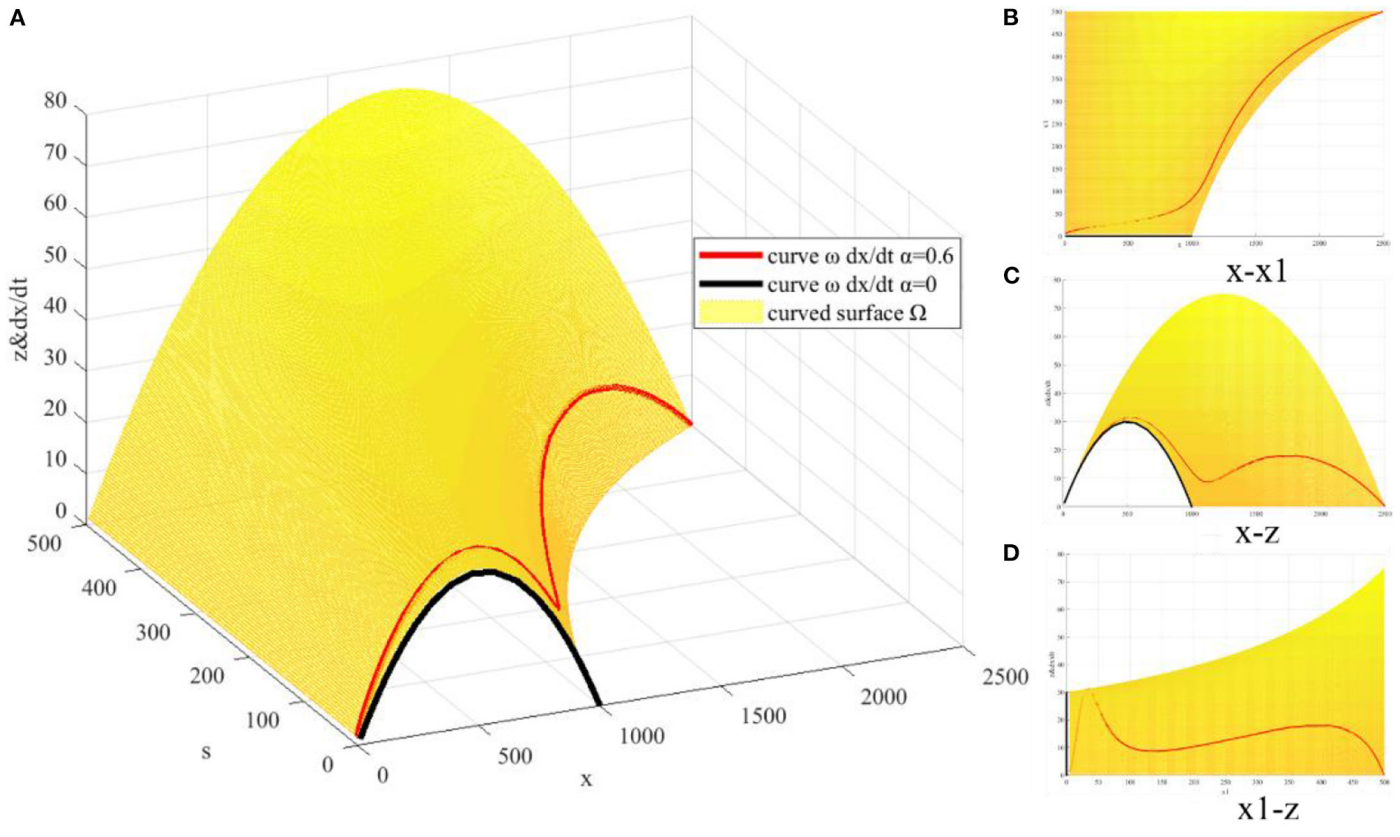
Model solution *x*(*t*) and $\frac{dx}{dt}$.

Let


(13) $$\begin{array}{l} z\left(x,s\right)=rx\left(1-\left(1-\alpha \frac{s}{K_{1}}\right)\frac{x}{K}\right) \end{array}$$

where $x\left(0\right)\leq x<\frac{K}{1-\alpha },\;s\left(0\right)\leq s<K_{1},z\geq 0$. It can be seen that *z* is a binary function of *x* and *s*, corresponding to a space surface Ω. When 0 ≤ *t* < +∞, $x(t)=\frac{x(0)\exp(rt)}{1+x(0)\int_{0}^{t}\frac{rexp(rv)}{g(v)}dv}$, $s\left(t\right)=\frac{K_{1}}{1+\left(\frac{K_{1}}{s\left(0\right)}-1\right)\exp\left(-r_{1}t\right)}$. So $\frac{dx}{dt}$ is essentially a space curve ω on the space surface Ω, whose shape is related to the parameter α. According to the set parameters, the corresponding images of space surface Ω and the change rate of COVID-19 vaccination quantity $\frac{dx}{dt}$(α = 0, α = 0.6) are drawn. It is observed that when α = 0, there is no effect of web search variable (Figure 4A). $\frac{dx}{dt}$ has an extreme point at $x=\frac{K}{2}=500$, corresponding to an inflection point *P*_1_(*T*_1_, 500) of *x*(*t*), where $T_{1}=\frac{1}{r}\ln\left(\frac{K}{x\left(0\right)}-1\right)=38.3$, and the coordinates of the inflection point *P*_1_(38.3, 500) are obtained. When α = 0.6, *s*(*t*) acts on *x*(*t*), and the web search variable has a significant promoting effect on the increase of the quantity of COVID-19 vaccination (Figure 4B), where the inflection point corresponding to *s*(*t*) is $P_{2}\left(T_{2},\frac{K_{1}}{2}\right)$,$T_{2}=\frac{1}{r_{1}}\ln\left(\frac{K_{1}}{s\left(0\right)}-1\right)=91.9$. The coordinates of the inflection point *P*_2_(91.9, 250) are obtained. *s*(*t*) leads to three extreme points of $\frac{dx}{dt}$ (Figure 4A), corresponding to the three inflection points of *x*(*t*), *P*_3_(39.0, 529.4), *P*_4_(72.4, 1120.1) and *P*_5_(119.1, 1749.7).

Under the action of the search quantity before the inflection point *P*_2_ of *s*(*t*), *P*_1_ becomes *P*_3_, but the time moves later, and the function value becomes larger (Figure 4C). *x*^′′^(*t*)>0 when on the left of *P*_3_ and *x*^′′^(*t*) < 0 when on the right of *P*_3_. The curve growth trend of *x*(*t*) changes from concave to convex. The first area of rapid growth in the quantity of COVID-19 vaccination is formed around *P*_3_, what plays a major role is the increasing of *x*(*t*) itself and the quantity of web search *s*(*t*) plays a minor role (Figure 4C). Under the action of the inflection point *P*_2_ of *s*(*t*), a new inflection point *P*_5_ is generated on *x*(*t*) curve. *P*_5_ lags behind *P*_2_, but the function value 1749.7 is obviously increased compared with (*K*+250), indicating that the effect of *s*(*t*) on *x*(*t*) requires a certain delay time, but stimulates a large function increment. In addition, like *P*_3_, the curve growth trend of *x*(*t*) changes from concave to convex, forming a second rapid growth area around *P*_5_, in which *s*(*t*) plays a major role while *x*(*t*) plays a minor role (Figure 4D). The inflection point *P*_4_ is just the node connecting these two rapid growth areas, where the curve growth trend of *x*(*t*) changes from convex to concave, and *P*_4_ is very close to the midpoint (79.1, 1139.5) of *P*_3_ and *P*_5_.

**Figure 5:**
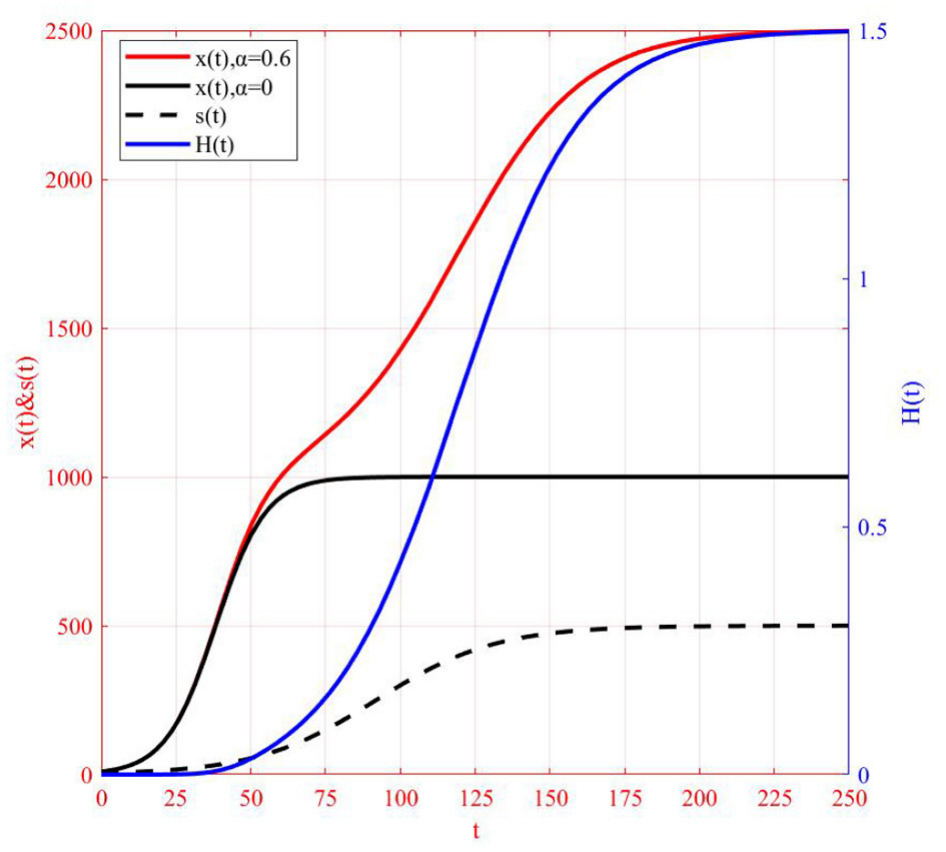
Comparison of model solution *x*(*t*) and *H*(*t*).

After clarifying the characteristics of the model solution, corresponding curves of *x*(*t*), *s*(*t*), and *H*(*t*) are drawn to further study the degree of eliminating COVID-19 vaccine hesitancy through web search (Figure 5). It is observed that *H*(*t*) increases monotonically from 0 to $H_{\infty }=\frac{\alpha }{1-\alpha }=1.5$, and its curve is an *S*-shaped curve. The coordinates of the inflection point of the curve are *P*_7_(119.2, 0.8), and the coordinates of the zero points of the third derivative are *P*_6_(90.5, 0.3) and *P*_8_(144.5, 1.2). It is observed that the zero points of the third derivative divide the whole curve into three parts, where the time difference between *P*_6_ and *P*_8_ is 54.0, and the increment of the elimination degree is 0.9, accounting for 57.2% of the total, indicating that it can be eliminated to a great extent in a short time. Therefore, the period **[**90.5, 144.5] has become the key time period for web search to eliminate COVID-19 vaccine hesitancy.

### 4.2. The effect of ***s***(***t***) on ***x*(*t*)**

**Figure 6:**
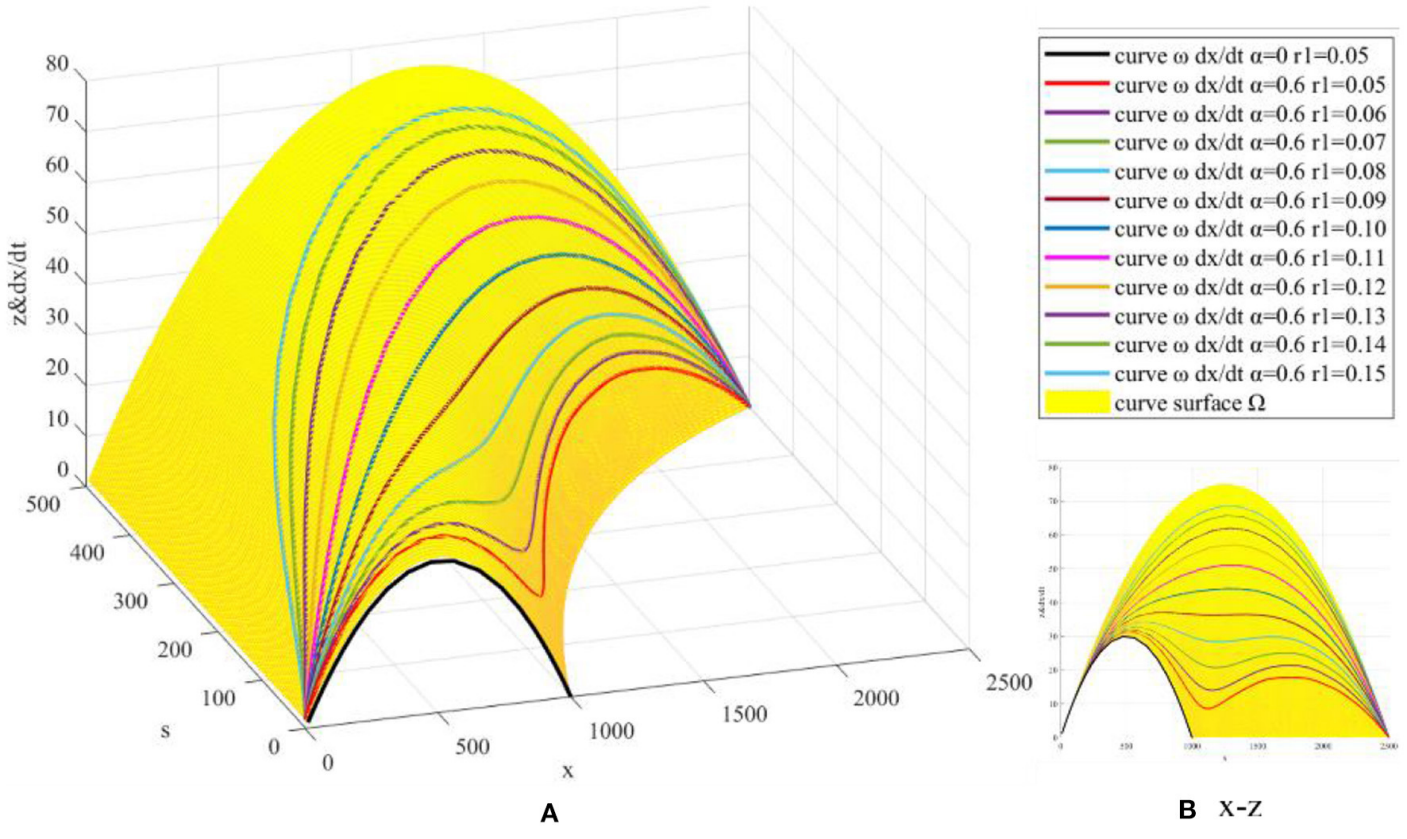
Simulation based on *r*_1_.

**Figure 7:**
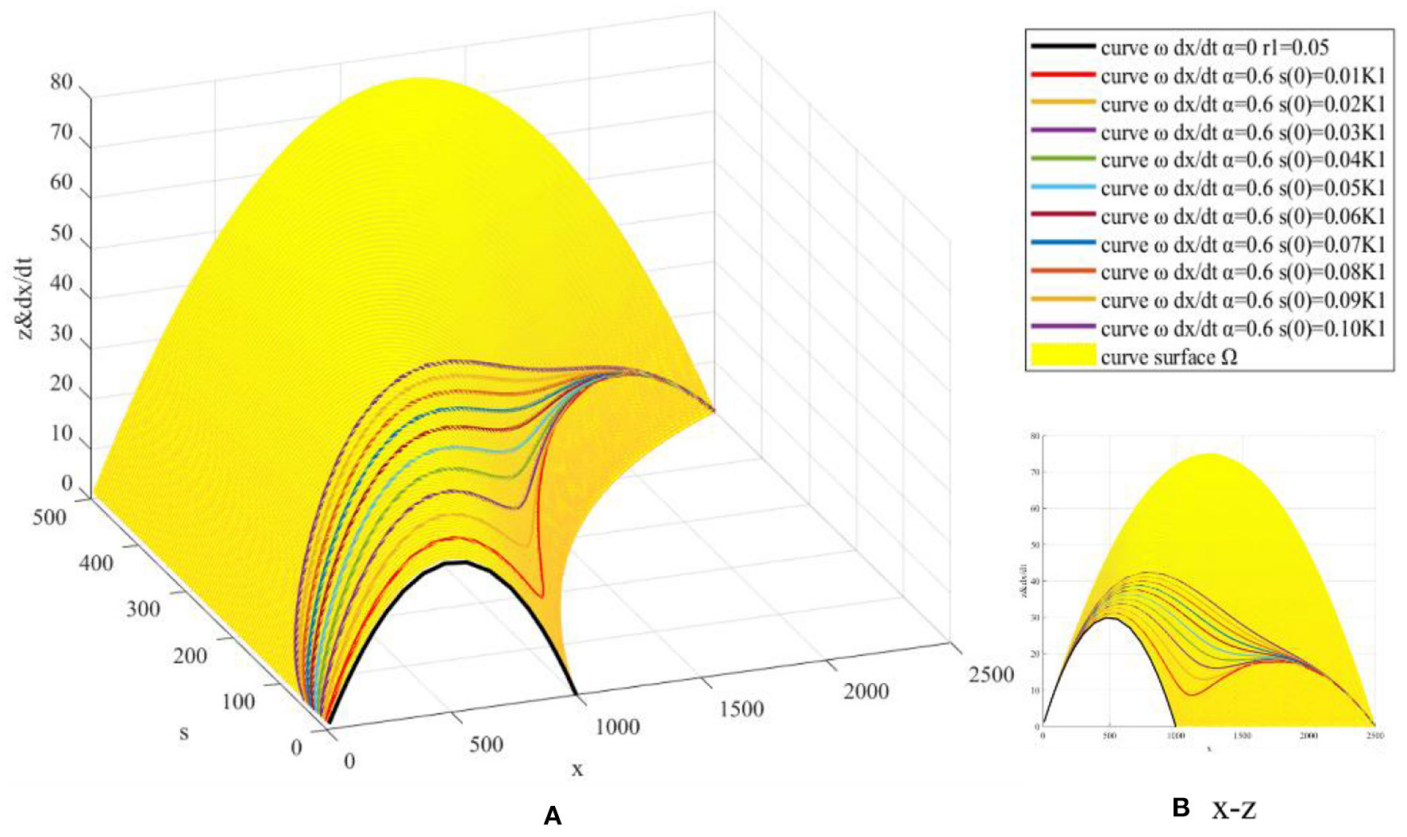
Simulation based on *s*(0).

The process parameters *r*_1_ and initial value parameter *s*(0) are simulated. ① Fix model parameters *r*, *K*, *x*(0), *K*_1_, *s*(0), and α, let *r*_1_ = 0.05:0.01:0.15, and draw *z*(*x, s*) surface and $\frac{dx}{dt}$ curve (Figure 6). ② Fix model parameters *r*, *K*, *x*(0), *K*_1_, *r*_1_, and α, let *s*(0) = 0.01*K*_1_:0.01*K*_1_:0.10*K*_1_, and draw *z*(*x, s*) surface and $\frac{dx}{dt}$ curve (Figure 7).

The growth rate *r*_1_ of *s*(*t*) represents the maximum growth rate of web search quantity, reflecting the intensity of netizens' independent search for COVID-19 vaccine. Through numerical simulation based on *r*_1_, it is found that with the increase of *r*_1_, the second maximum point corresponding to $\frac{dx}{dt}$ gradually increases. As a result, the distance between the two maximum points of the *x*(*t*) curve gradually decreases, and the three extreme points converge into one extreme point (Figure 6B), which indicates that *x*(*t*) gradually changes from a double *S*-shaped curve to a single *S*-shaped curve and will increase rapidly in a short time (the time difference of the third derivative zero points corresponding to the single *S*-shaped curve). On the contrary, when *r*_1_ decreases, it will delay the growth rate of *x*(*t* ).

In addition, $\frac{dx}{dt}$ increases with the increase of *r*_1_, and web search can effectively eliminate vaccine hesitancy. In this process, young people have more access to the Internet and elderly people have less access to the Internet. Therefore, the web search growth rate of young people is higher than the growth rate *r*_1_ in the model and the web search growth rate of elderly people is lower than the growth rate *r*_1_ in the model. In order to eliminate the vaccine hesitancy of the elderly population, according to the model in this paper, it will be good countermeasures to directly or indirectly increase the growth rate of web search. For example, the government should promote the construction of Internet infrastructure, help the elderly people to use mobile phones and other Internet access devices, and develop more intelligent and user-friendly Internet products for the elderly. At the same time, the government should carry out the COVID-19 vaccination publicity in an all-round and multiform way, further improve the awareness rate of the elderly on vaccination, so as to indirectly promote their web search for vaccination.

Initial value *s*(0) represents the initial state of netizens' independent search for COVID-19 vaccine in a certain evolution cycle. Through numerical simulation based on *s*(0), it is found that with the increase of *s*(0), the first maximum point corresponding to $\frac{dx}{dt}$ gradually increases (Figure 7B), resulting in the convergence of three extreme points into one extreme point and *x*(*t*) will increase rapidly in a short time. This is different from the process parameter *r*_1_. *s*(0) mainly acts on the first half of *x*(*t*) curve, while *r*_1_ mainly acts on the second half of *x*(*t*) curve. By comparing the images, it can be found that the effect of *r*_1_ on *x*(*t*) is significantly stronger than *s*(0), mainly because *x*(*t*) is smaller in the first half (*x*<*K*)and larger in the second half (*x*>*K*).

In addition, the zero point formulas of the third derivative for *s*(*t*) are $\frac{1}{r_{1}}\ln\left(\frac{\frac{K_{1}}{s\left(0\right)}-1}{2+\sqrt{3}}\right)$ and $\frac{1}{r_{1}}\ln\left(\frac{\frac{K_{1}}{s\left(0\right)}-1}{2-\sqrt{3}}\right)$, and the time difference between the zero points is $\frac{1}{r_{1}}\ln\left(2+\sqrt{3}\right)$. With the increase of growth rate *r*_1_ and initial value *s*(0), the third derivative zero points are advanced and the corresponding time difference is shortened. *s*(*t*) will approach *K*_1_ rapidly in a short time, leading to a rapid increase of *x*(*t*) in a short time, increasing the possibility of *s*(*t*) breaking the upper limit *K*_1_ of the model, and making *x*(*t*) enter a new evolution cycle. Therefore, how to stimulate netizens' willingness to carry out web search for COVID-19 vaccine independently and improve the maximum growth rate and initial value of web search quantity are important strategies to eliminate COVID-19 vaccine hesitancy.

### 4.3. Simulation based on stationary point parameter **α**

**Figure 8:**
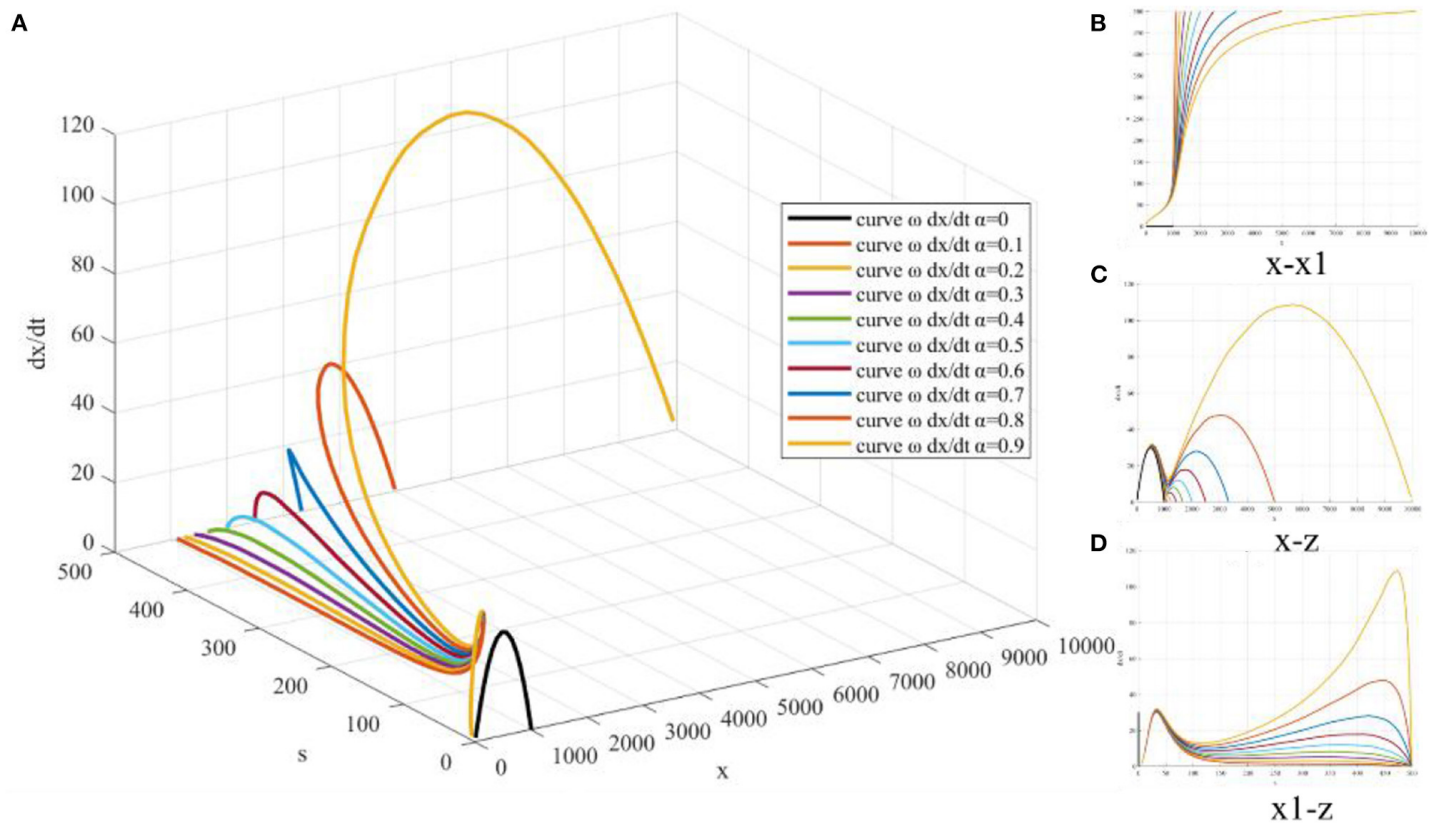
Simulation based on stationary point parameter α.

Fix model parameters *r*, *K*, *x*(0), *r*_1_, *K*_1_, *s*(0), and take α = 0.1:0.1:0.9 to draw surface *z*(*x, s*) and curve $\frac{dx}{dt}$ (Figure 8). The promoting coefficient α represents the degree that the quantity of web search transformed into the quantity of COVID-19 vaccination. Through numerical simulation based on stationary point parameter α, it is found that with the increase of α, the upper limit $\frac{K}{1-\alpha }$ of *x*(*t*) and the elimination rate $H_{\infty }=\frac{\alpha }{1-\alpha }$ are increased. Therefore, the higher the degree of transforming the quantity of web search into the quantity of COVID-19 vaccination, the better the effect of eliminating COVID-19 vaccine hesitancy. The curve corresponding to $\frac{dx}{dt}$ still has three extreme points, but the change of the second maximum point is proportional to the parameter α and gradually moves backward with the increase of α, so the *x*(*t*) curve is still a double *S*-shaped structure. An obvious area of rapid growth of the quantity of COVID-19 vaccination will be formed around the second maximum point, and the degree of quantity growth is much higher than the rapid growth area formed around the first maximum point. Therefore, promoting the web search of netizens, setting the agenda according to the web search problems and their recommended contents, and actively eliminating the hesitation of netizens will effectively improve the promoting coefficient and make the quantity of web search transform into more COVID-19 vaccination.

## 5. Empirical analysis

### 5.1. Data sources

**Figure 9:**
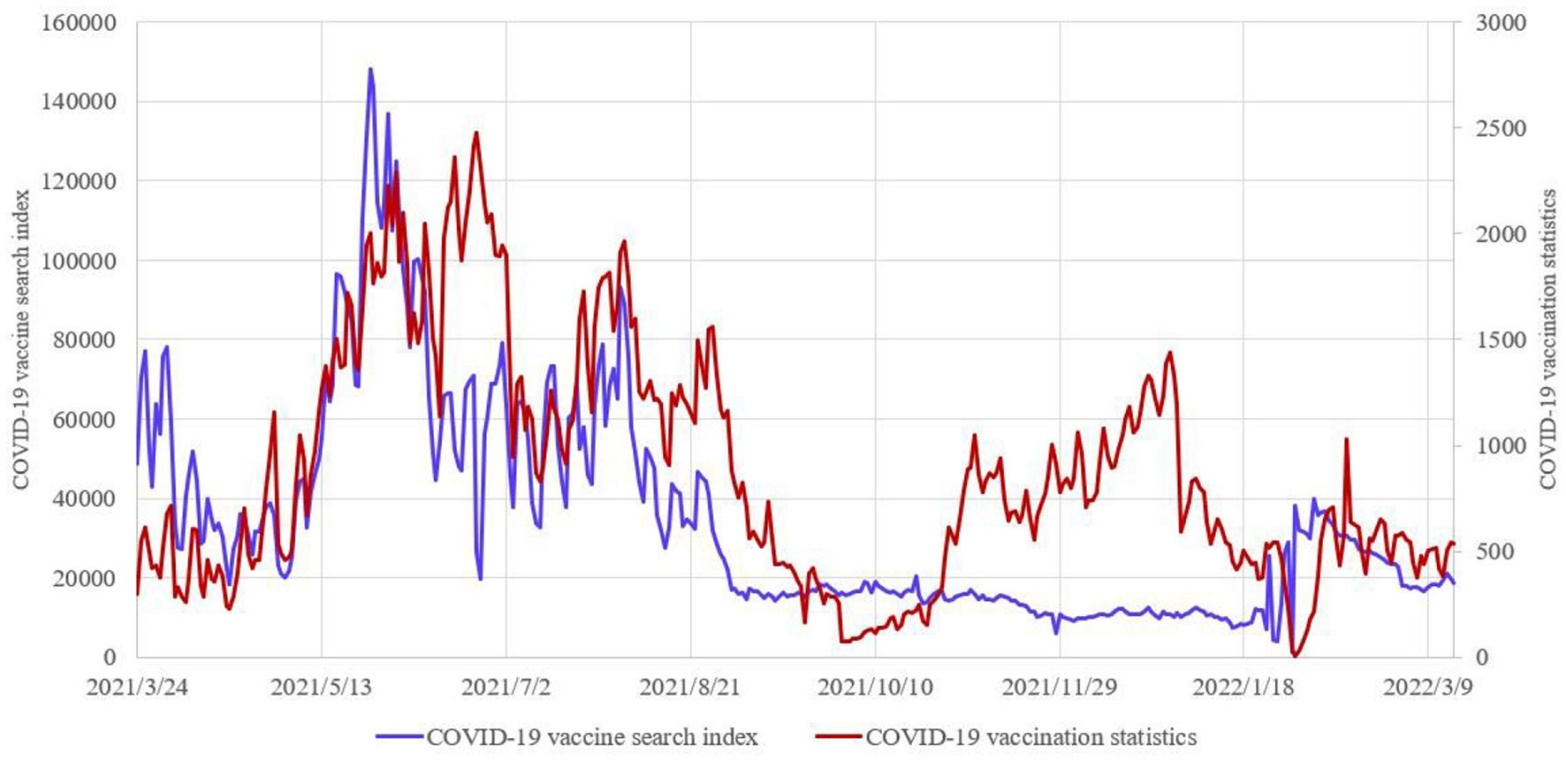
Statistics of COVID-19 vaccination and Baidu search.

To verify the rationality of the modeling in this paper, we select the vaccination data of COVID-19 (unit: 10,000 doses) and Baidu search data corresponding to COVID-19 vaccine from 2021.3.24 to 2022.3.11 as the modeling data (Figure 9). Based on the search volume of netizens in Baidu and taking keywords as the statistical object, Baidu search data are obtained by scientifically analyzing and calculating the weighted search frequency of each keyword in Baidu web search.

### 5.2. Modeling verification

#### 5.2.1. Macro data modeling

In order to verify the consistency between the model and the real data in macro-range. Change model (4) into its corresponding difference equation.


$$\{\begin{array}{c} \Delta x\left(k\right)=rx-\frac{r}{K}x^{2}+\frac{r\alpha }{K_{1}K}sx^{2}\\ \Delta s\left(k\right)=r_{1}s-\frac{r_{1}}{K_{1}}s^{2} \end{array}$$

According to the COVID-19 vaccination data and the web search data from 2021.3.24 to 2022.3.11, the model parameters are determined by regression analysis. First of all, for $\Delta s\left(k\right)=r_{1}-\frac{r_{1}}{K_{1}}s^{2},\;r_{1}=0.02,-\frac{r_{1}}{K_{1}}=-1.8\times 10^{-9}$ are obtained by regression analysis, where the coefficient of determination *R*^2^ = 0.70, and the *P*-values corresponding to the two regression coefficients are 7.56 × 10^−77^ and 2.89 × 10^−65^, respectively. The results of regression analysis are significant. Secondly, for $\Delta x\left(k\right)=rx-\frac{r}{K}x^{2}+\frac{r\alpha }{K_{1}K}sx^{2}$, $r=0.04,-\frac{r}{K}=-3.2\times 10^{-7},\frac{r\alpha }{K_{1}K}=1.7\times 10^{-14}\;$are obtained through regression analysis, where the coefficient of determination *R*^2^ = 0.78, and the corresponding *P-*values of the three regression coefficients are 1.23 × 10^−60^,1.93 × 10^−36^,1.04 × 10^−27^, respectively. The results of regression analysis are significant. Based on the above calculation results, we can obtain the model parameters through simple calculation, *r* = 0.04, *K* = 116061.9, *r*_1_ = 0.02, *K*_1_ = 11673560.4, α = 0.6.

**Figure 10:**
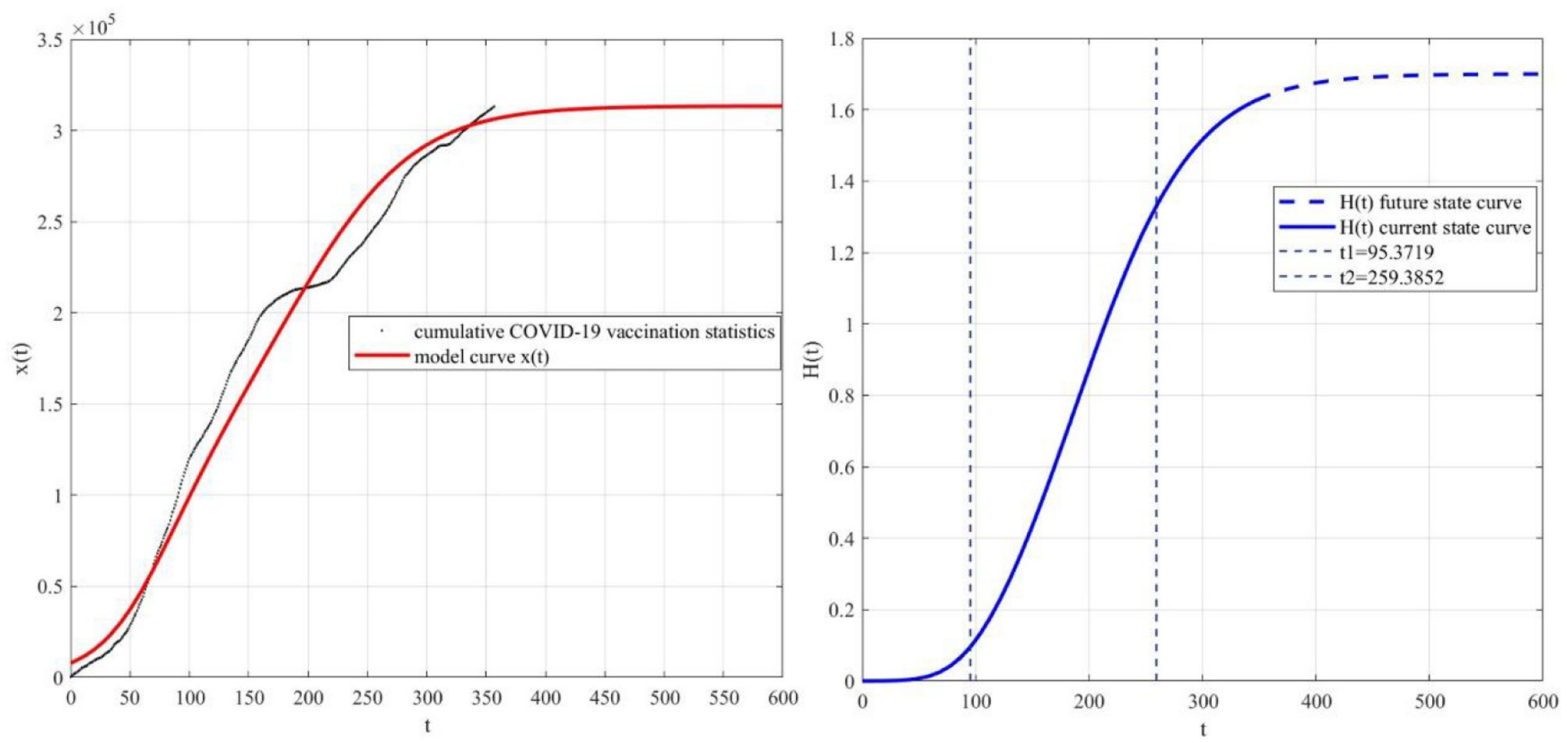
Real data, *x*(*t*) and *H*(*t*).

Take the average value of the first 10% of the real data as the initial value of the model, draw the comparison diagram of the real data and the modeling curve, and the curve of the elimination function *H*(*t*) (Figure 10). The zero point coordinates of the third derivative corresponding to the elimination function *H*(*t*) are calculated as (95.4, 0.1) and (259.4, 1.3). It is found that the modeling curve is basically consistent with the evolutionary trend of real data, which verifies the rationality of the model constructed in this paper. The optimal range of COVID-19 vaccine hesitancy elimination is [95.4, 259.4], the increment of *H*(*t*) is 72.7%, and the elimination rate of COVID-19 vaccine hesitancy elimination through web search is *H*_∞_ = 1.7.

#### 5.2.2. Micro data modeling

**Figure 11:**
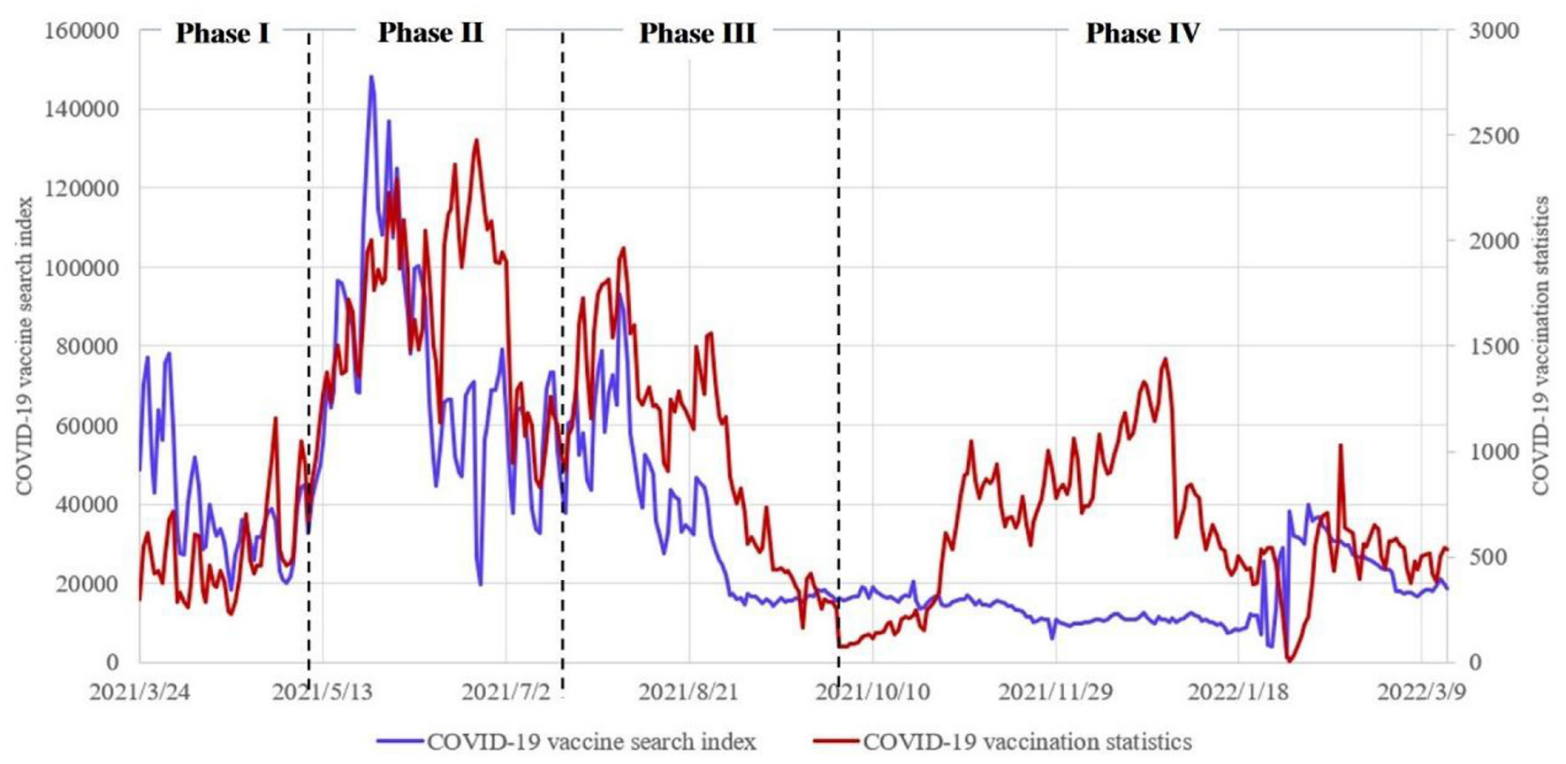
Segmentation of statistical data curve.

According to the observation of the statistical data curve, it is found that the changes of vaccination data and web search data show obvious periodicity. In order to further verify the consistency between the model and the real data in micro-range, the entire data range is divided into four parts (Figure 11) according to the periodicity of web search data (*S*-shaped), and data modeling is carried out to verify the applicability of the model in the micro-range.

Data modeling is carried out based on vaccination data and web search data in four stages, and the model parameters and fitting effect data corresponding to the four stages are obtained through regression analysis (Table 2). It is found that the data fitting effect is very good in the four stages, and the regression analysis results are significant, which shows that the model is in good agreement with the real data in the micro-range. The dynamic model of COVID-19 vaccination quantity change under the effect of web search can be applied to analyze the problem of eliminating vaccine hesitancy through web search.

**Table 2:** Segmented data modeling.

**Stage (start and end time)**	**Number of data**	**Model parameters**	**Fitting coefficient R^2^**	**Fitting *p*-value**	**H_∞_**
Phase I (2021.3.24–2021.5.2)	40*2	*r*_1_ = 0.11 *K*_1_ = 1723605.6	0.75	8.60 × 10^−9^ 3.07 × 10^−6^	4.3
		*r* = 0.27 *K* = 5686.9 α = 0.8	0.88	4.35 × 10^−8^ 3.22 × 10^−6^ 5.99 × 10^−6^	
Phase II (2021.5.3–2021.7.16)	75*2	*r*_1_ = 0.07 *K*_1_ = 5463326.3	0.85	6.24 × 10^−24^ 2.89 × 10^−18^	0.6
		*r* = 0.08 *K* = 82201.9 α = 0.4	0.94	3.40 × 10^−19^ 8.20 × 10^−6^ 2.45 × 10^−2^	
Phase III (2021.7.17–2021.9.30)	76*2	*r*_1_ = 0.09 *K*_1_ = 2683296.7	0.80	3.42 × 10^−23^ 8.51 × 10^−20^	1.0
		*r* = 0.13 *K* = 40100.3 α = 0.5	0.91	1.30 × 10^−21^ 1.07 × 10^−11^ 4.59 × 10^−7^	
Phase IV (2021.10.1–2022.3.11)	167*2	*r*_1_ = 0.02 *K*_1_ = 4954840.5	0.79	5.63 × 10^−24^ 2.17 × 10^−6^	2.6
		*r* = 0.06 *K* = 66492.0 α = 0.7	0.92	2.50 × 10^−68^ 5.13 × 10^−48^ 3.86 × 10^−26^	

### 5.3. Apply the model to carry out prediction

**Figure 12:**
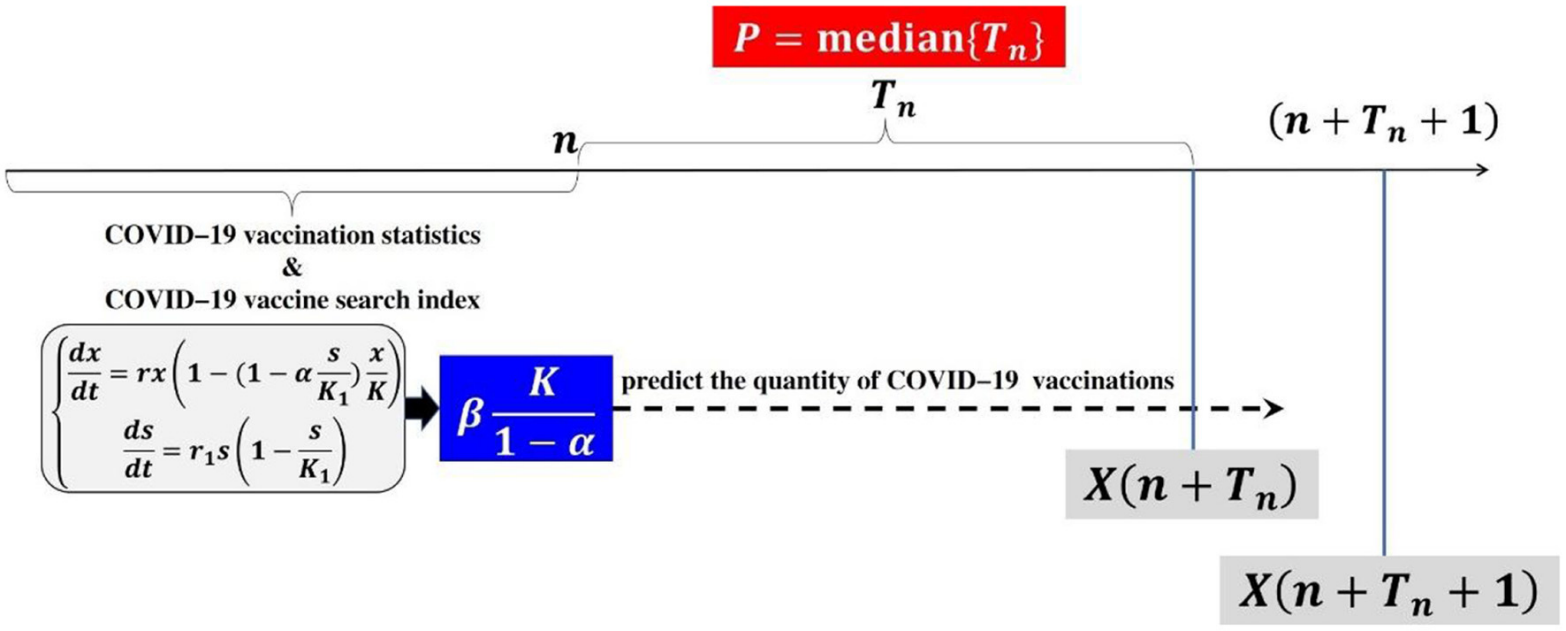
Prediction ideas.

Through the previous empirical analysis, the model constructed in this paper is in good agreement with the real data, but for policy makers, they want to predict the future situation based on the current data, and then put forward better countermeasures and suggestions for COVID-19 vaccination. Firstly, the model parameters are determined through the current web search data and vaccination data, and then the future trend is predicted according to the equilibrium point $\frac{K}{1-\alpha }$, so as to study the predictive ability of the model. Based on the first *n* groups of web search and COVID-19 vaccination data, dynamic prediction is carried out by adding new data, and the equilibrium point $\frac{K}{1-\alpha }$ is calculated to predict the future trend. When $X\left(n+T_{n}\right)\leq \beta \frac{K}{1-\alpha }<X\left(n+T_{n}+1\right)$, the prediction length of the model is defined as *T*_*n*_, the median of *T*_*n*_ is *P* = *median*{*T*_*n*_}, where *X*(*n*) represents the real data, *n*≥1, integer *T*_*n*_≥0, and β represents the prediction floating value, generally taking β = 1+5%. When it is determined that the model parameters do not conform the model conditions or the *P-*value of regression analysis is large (*P*>0.05) or *T*_*n*_ = 0, the dynamic prediction will be stopped. The prediction ideas are shown in Figure 12.

Data modeling is carried out based on the first stage data (2021.3.24–2021.5.2), and the first stage prediction data table is obtained (Table 3). When *n* = 52, α = 1.01>1 does not conform the model conditions, so the dynamic prediction is stopped. The observation of prediction results show that the dynamic prediction based on the first stage data has a significant effect of regression analysis. The prediction length *T*_*n*_ is between 11 and 84, *P* = 16.5, and the prediction length can basically cover the second stage data.

**Table 3:** Prediction data of the first stage.

**Time**	**Starting point n**	**Equilibrium point $\beta \frac{\text{K}}{1-\alpha }$**	**Length T_n_**	***P*-value of regression analysis**	***R* ^2^**
2021.3.24–2021.5.2	40	31,628.4	13	8.56 × 10^−9^; 3.07 × 10^−6^; 4.35 × 10^−8^; 3.22 × 10^−6^; 5.99 × 10^−6^	0.88 0.75
2021.3.24–2021.5.3	41	31,524.4	12	3.93 × 10^−9^; 1.53 × 10^−6^; 3.74 × 10^−7^; 3.00 × 10^−5^; 5.99 × 10^−5^	0.86 0.75
2021.3.24–2021.5.4	42	31,799.2	11	2.03 × 10^−9^; 8.72 × 10^−7^; 1.64 × 10^−6^; 1.44 × 10^−4^; 3.00 × 10^−4^	0.85 0.75
2021.3.24–2021.5.5	43	32,559.0	11	1.29 × 10^−9^; 6.19 × 10^−7^; 4.55 × 10^−6^; 4.48 × 10^−4^; 9.64 × 10^−4^	0.85 0.74
2021.3.24–2021.5.6	44	36,072.5	12	2.04 × 10^−9^; 1.16 × 10^−6^; 1.85 × 10^−6^; 2.37 × 10^−4^; 5.07 × 10^−4^	0.85 0.74
2021.3.24–2021.5.7	45	42,263.7	15	4.27 × 10^−9^; 2.92 × 10^−6^; 2.56 × 10^−7^; 4.14 × 10^−5^; 8.24 × 10^−5^	0.87 0.73
2021.3.24–2021.5.8	46	49,629.4	18	8.50 × 10^−9^; 7.00 × 10^−6^; 2.14 × 10^−7^; 4.75 × 10^−5^; 9.15 × 10^−5^	0.87 0.72
2021.3.24–2021.5.9	47	51,311.8	18	6.71 × 10^−9^; 6.68 × 10^−6^; 2.09 × 10^−6^; 5.51 × 10^−4^; 1.10 × 10^−3^	0.86 0.72
2021.3.24–2021.5.10	48	57,898.7	21	9.06 × 10^−9^; 1.11 × 10^−5^; 2.21 × 10^−6^; 8.36 × 10^−4^; 1.68 × 10^−3^	0.86 0.72
2021.3.24–2021.5.11	49	69,356.4	25	1.51 × 10^−8^; 2.27 × 10^−5^; 1.29 × 10^−6^; 7.47 × 10^−4^; 1.49 × 10^−3^	0.84 0.71
2021.3.24–2021.5.12	50	94,186.8	38	2.83 × 10^−8^; 5.27 × 10^−5^; 3.35 × 10^−7^; 2.95 × 10^−4^; 5.50 × 10^−4^	0.85 0.71
2021.3.24–2021.5.13	51	167,204.5	84	6.60 × 10^−8^; 1.50 × 10^−4^; 9.70 × 10^−8^; 1.24 × 10^−4^; 2.06 × 10^−4^	0.89 0.71

**Figure 13:**
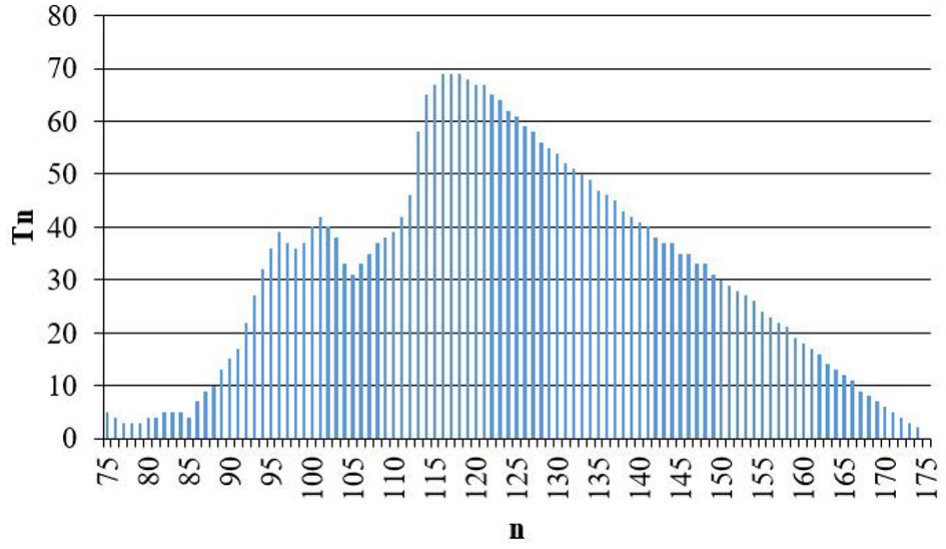
Prediction length of the second stage.

According to the above prediction ideas, continue to carry out dynamic prediction based on the second stage data (2021.5.3–2021.7.16), get the second stage prediction data, and draw the prediction length series {*T*_*n*_} curve (Figure 13). When *n* = 175, obtained that *T*_*n*_ = 0, so the dynamic prediction is stopped. By analyzing the prediction results, the dynamic prediction based on the second stage data shows significant regression analysis effect. The prediction length *T*_*n*_ is between 1 and 69, *P* = 33.

The prediction length can cover most of the third stage data, and the prediction persistence effect is significantly better than that based on the first stage data. The main reason is that the data fitting effect of the second stage is significantly better than that of the first stage. The dynamic prediction based on the above two stages has shown that the dynamic model has certain medium-term prediction ability.

**Figure 14:**
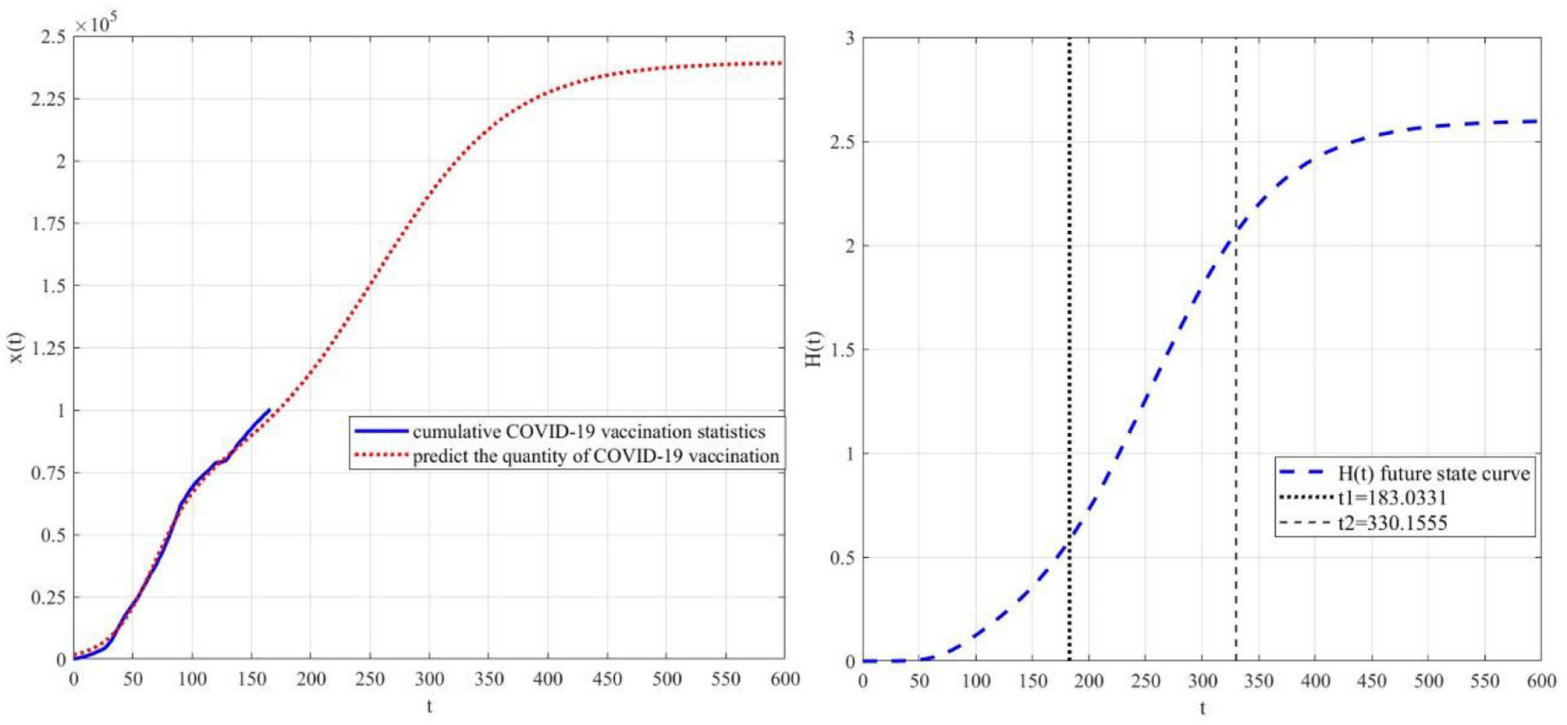
Prediction based on the fourth stage.

Finally, data modeling is carried out based on the fourth stage data (2021.10.1–2022.3.11), then the specific parameters *r*_1_ = 0.02, *K*_1_ = 4954840.5, *r* = 0.06, *K* = 66492.0, α = 0.7 are obtained. Based on this, the prediction curve and the elimination function prediction curve are obtained (Figure 14). It can be inferred that the quantity of COVID-19 vaccination will approach 239,421.9 in the recent evolution cycle. During the period of 2022.4.1–2022.8.26, the degree of eliminating COVID-19 vaccine hesitancy through web search will increase significantly (about 56.8%).

## 6. Conclusion

(1) This research analyzes the general process of eliminating COVID-19 vaccine hesitancy through web search, selects the COVID-19 vaccination quantity as the core variable, and constructs the dynamic model of COVID-19 vaccination quantity change under the effect of web search. In order to measure the degree of eliminating COVID-19 vaccine hesitancy through web search, we define the elimination function and elimination rate, and explain the dynamic mechanism of eliminating vaccine hesitancy and promoting the increase of COVID-19 vaccination quantity through web search.
(2) Numerical simulations are carried out for the dynamic model of COVID-19 vaccination quantity change under the effect of web search. We study characteristics of the model solution, the effect of process parameters and stationary point parameters on the quantity of COVID-19 vaccination and elimination of COVID-19 vaccine hesitancy, and explore the time nodes of the variable evolution. Then get the strategies to eliminate COVID-19 vaccine hesitancy, such as stimulating the willingness of netizens to independently carry out web search and actively responding to netizens' search problems. In addition, we believe that the method of promoting netizens' active search and voluntary vaccination will be more helpful to fundamentally eliminate vaccine hesitancy. For COVID-19, there are usually multiple doses of continuous vaccination. This method also provides a good basis for subsequent vaccination.
(3) This research studies the parameter estimation method of the dynamic model. Through the real data of COVID-19 vaccination and web search, the data modeling is carried out, and the regression analysis results are significant, which verifies the feasibility of the model. This model is applied to the prediction of the quantity of COVID-19 vaccination, and the results show that this method has a certain ability of medium-term prediction. The research on prediction problems can help to make vaccination plans and provide predictive suggestions for the dynamic adjustment of public health policies.

## Data availability statement

The original contributions presented in the study are included in the article/Supplementary material, further inquiries can be directed to the corresponding author.

## Author contributions

YX: conceptualization, validation, investigation, formal analysis, and writing—original draft. QL: writing—editing and data curation. WJ: data curation, investigation, and writing—editing. YL: conceptualization, funding acquisition, supervision, methodology, and writing—review. All authors contributed to the article and approved the submitted version.
