# Supplementary material for: Dynamic mechanism of eliminating COVID-19 vaccine hesitancy through web search
Source: Front Public Health. 2023 Jan 30;11:1018378. doi: 10.3389/fpubh.2023.1018378 (PMC9922755; doi:10.3389/fpubh.2023.1018378)
Supplement: Supplementary file 1 [file Table_1.DOCX]

1.data

1.1 COVID-19 vaccine - Baidu search index

Data from baidu search, https://index.baidu.com/v2/index.html#/, index word for "new vaccine", time for 2021/3/23-2022/3/11

1.2 COVID-19 vaccine - Vaccination data

Data from the national health committee of the People's Republic of China's official website, http://www.nhc.gov.cn/xcs/yqfkdt/gzbd_index.shtml, starting and ending time for 2021/3/23-2022/3/11 data

2. Procedures

2.1 The matlab plotting functions applied in the simulation diagram section

The matlab plotting functions applied in the simulation plot section

The initial values of x(1) and x(2) are 0.01k, and the initial values of x(3) are 0.01k1

function s= yimiao2022(t,x)

K=1000;K1=500;r=0.12;a=0.6;r1=0.05;

s=[r*x(1)*(1-x(1)/K),r*x(2)*(1-x(2)/K+a*(x(3)/K1)*(x(2)/K)),r1*x(3)*(1-x(3)/K1)]';

end

2.2 The matlab plotting function applied in the empirical part

The average of the first 10% of the real data is taken as the initial value of the model

function s= yimiao2022(t,x)

K=116061.8926;K1=11673560.4285;r=0.0366;a=0.6296;r1=0.0214;

s=[r*x(1)*(1-x(1)/K),r*x(2)*(1-x(2)/K+a*(x(3)/K1)*(x(2)/K)),r1*x(3)*(1-x(3)/K1)]';

end

function s= yimiao2022(t,x)

K=66492.0253;K1=4954840.4881;r=0.0556;a=0.7223;r1=0.0179;

s=[r*x(1)*(1-x(1)/K),r*x(2)*(1-x(2)/K+a*(x(3)/K1)*(x(2)/K)),r1*x(3)*(1-x(3)/K1)]';

end
